# A Protocol to Investigate Deep Brain Stimulation for Refractory Tinnitus: From Rat Model to the Set-Up of a Human Pilot Study

**DOI:** 10.3390/audiolres13010005

**Published:** 2022-12-31

**Authors:** Gusta van Zwieten, Jana V. P. Devos, Sonja A. Kotz, Linda Ackermans, Pia Brinkmann, Lobke Dauven, Erwin L. J. George, A. Miranda L. Janssen, Bernd Kremer, Carsten Leue, Michael Schwartze, Yasin Temel, Jasper V. Smit, Marcus L. F. Janssen

**Affiliations:** 1 School for Mental Health and Neuroscience (MHeNS), Maastricht University, 6229 ER Maastricht, The Netherlands; 2 Department of Neurosurgery, Maastricht University Medical Center, 6229 HX Maastricht, The Netherlands; 3 Department of Ear Nose and Throat/Head and Neck Surgery, Maastricht University Medical Center, 6229 HX Maastricht, The Netherlands; 4 Faculty of Psychology and Neuroscience, Department of Neuropsychology & Psychopharmacology, Maastricht University, 6229 ER Maastricht, The Netherlands; 5 Department of Methodology and Statistics, School for Public Health and Primary Care, Maastricht University, 6229 HA Maastricht, The Netherlands; 6 Department of Psychiatry and Psychology, Maastricht University Medical Center, 6229 HX Maastricht, The Netherlands; 7 Department of Ear Nose and Throat/Head and Neck Surgery, Zuyderland, 6419 PC Heerlen, The Netherlands; 8 Department of Clinical Neurophysiology, Maastricht University Medical Center, 6229 HX Maastricht, The Netherlands

**Keywords:** deep brain stimulation, tinnitus, MGB, auditory thalamus, randomized controlled trial

## Abstract

Background: Chronic tinnitus can have an immense impact on quality of life. Despite recent treatment advances, many tinnitus patients remain refractory to them. Preclinical and clinical evidence suggests that deep brain stimulation (DBS) is a promising treatment to suppress tinnitus. In rats, it has been shown in multiple regions of the auditory pathway that DBS can have an alleviating effect on tinnitus. The thalamic medial geniculate body (MGB) takes a key position in the tinnitus network, shows pathophysiological hallmarks of tinnitus, and is readily accessible using stereotaxy. Here, a protocol is described to evaluate the safety and test the therapeutic effects of DBS in the MGB in severe tinnitus sufferers. Methods: Bilateral DBS of the MGB will be applied in a future study in six patients with severe and refractory tinnitus. A double-blinded, randomized 2 × 2 crossover design (stimulation ON and OFF) will be applied, followed by a period of six months of open-label follow-up. The primary focus is to assess safety and feasibility (acceptability). Secondary outcomes assess a potential treatment effect and include tinnitus severity measured by the Tinnitus Functional Index (TFI), tinnitus loudness and distress, hearing, cognitive and psychological functions, quality of life, and neurophysiological characteristics. Discussion: This protocol carefully balances risks and benefits and takes ethical considerations into account. This study will explore the safety and feasibility of DBS in severe refractory tinnitus, through extensive assessment of clinical and neurophysiological outcome measures. Additionally, important insights into the underlying mechanism of tinnitus and hearing function might be revealed. Trial registration: ClinicalTrials.gov NCT03976908 (6 June 2019).

## 1. Introduction

Tinnitus, commonly known as “ringing of the ears”, is one of the largest health challenges in the world [1]. According to a recent large survey, approximately 6.4% of Americans experience persistent tinnitus [2]. One in ten patients experiences the most extreme and debilitating form of tinnitus. Sleep deprivation, anxiety, and depression often accompany tinnitus and severely affect the patient’s quality of life [3,4,5]. In turn, this places a huge burden on society, and healthcare costs, and decreases productivity [6]. 

Subjective tinnitus has a multifactorial origin with heterogeneous patient profiles, which makes it a highly complex condition. The absence of an underlying medical cause in most cases leaves many patients without an available curative evidence-based treatment [7,8]. Tinnitus combined with sensorineural hearing loss might benefit from hearing aids. However, somewhere between 22% and 80% of affected patients are adequately served by using hearing aids [9,10]. The current clinical practice primarily aims at reducing the impact of tinnitus by providing psychoeducation and improving coping strategies via various psychological interventions [11,12]. 

The etiological and pathophysiological mechanisms of subjective tinnitus are complex and not fully understood. Many investigators feel that in nearly all tinnitus cases, there is some degree of cochlear impairment, leading to diminished auditory nerve activity reaching the cochlear nuclei [13]. Much evidence implicates the head and neck somatosensory system as a separate major factor in the development of tinnitus. It is likely that most tinnitus develops as a result of interactions between these two systems within the central nervous system [14]. According to current theories, tinnitus is associated with increased neural activity in auditory cortices, possibly resulting from maladaptive gating [15,16] and/or an increase in central gain [17]. Specific neural correlates described in tinnitus models are enhanced neuronal synchrony, increased spontaneous firing, and changes in tonotopic organization [18]. 

A commonly applied neuromodulation technique is deep brain stimulation (DBS). This therapy has been widely used in neurologic and neuropsychiatric disorders such as Parkinson’s disease. DBS is generally applied using high-frequency stimulation (>100 Hz), to disrupt pathological neuronal activity and oscillations [19,20]. Hypothetically, this results in an alteration of tinnitus perception and related distress. Further, patients treated with DBS for a movement disorder sometimes also suffered from tinnitus. DBS of non-auditory structures in these patients led to diminished or completely suppressed tinnitus [21,22,23,24,25,26,27]. Other implants that could potentially influence tinnitus, and have been investigated, are a cochlear implant (CI) [28] and an auditory brainstem implant (ABI) [29], however, these can only be used in patients with severe hearing loss. In addition, other neuromodulation techniques, such as vagal nerve stimulation [30] and cortical stimulation [31], have been investigated with various degrees of success. These latter treatments can also be used in patients without hearing loss, however, at the moment, there is insufficient evidence to implement these treatments in clinical practice. For a comprehensive review of neuromodulation for tinnitus, see Deklerck et al. [32]. Our hypothesis is that influencing the pathological tinnitus network at any level could be beneficial in theory, but the site at which stimulation is performed strongly influences the outcome. Stimulation in close proximity to the site at which the pathological activity is generated, i.e., within the brainstem [33], might be a more direct and thus efficient target. This could also explain why non-invasive cortical stimulation fails to show conclusive favorable outcomes [34,35].

Preclinically, at multiple levels of the central auditory pathway from cochlear nuclei to the auditory cortex, tinnitus-related neuronal activity is similar to subthalamic nucleus activity in Parkinson’s disease, i.e., enhanced spontaneous activity and burst firing [36,37,38,39]. The primary role of auditory thalamic neurons is to actively and dynamically shape neural representations of information and to control which information reaches the cerebral cortex [40].

Moreover, preclinical studies support the beneficial effects of DBS on tinnitus when applied in auditory brain areas [21,22,23,24,25,26,27]. In Table 1 we listed all currently available animal and human studies that applied DBS for tinnitus. In our lab a rat model for tinnitus was used in order to test DBS as a potential treatment for tinnitus. Noise exposure was used to induce tinnitus in rats after which a gap-prepulse inhibition of the acoustic startle reflex (GPIAS) also known as a gap detection task [41] established the presence of tinnitus. This task exploits the acoustic startle reflex which is present in all mammals and consists of a contraction of the major muscles in response to an unexpected loud noise [42]. A reduction in tinnitus-like behavior was shown when DBS was applied in several structures along the classical auditory pathway, including the dorsal cochlear nucleus [43,44,45], inferior colliculus [46], and medial geniculate body (MGB) [47]. Importantly, no undesired side effects occurred. DBS of the MGB did not lead to anxiety or disturbed locomotor activity. DBS of the inferior colliculi did not cause any detectable hearing impairment [48]. Other groups also showed beneficial effects in tinnitus behavior in animal rat models, in the caudate [45] and the dorsal cochlear nucleus [44,45]. A similar setup was used as in our studies. These results also indicate that (high-frequency) stimulation anywhere within the pathological tinnitus network could have a beneficial effect on tinnitus [49,50,51]. 

The MGB is a preferred target area as opposed to other auditory subcortical structures [50], as the auditory thalamus is readily accessible in stereotactic surgery. Consequently, targeting the auditory thalamus bears smaller surgical risks and complications such as bleeding and potential neurological deficit. The MGB of the thalamus is a major relay and gateway between the midbrain and cortex, and a core structure in tinnitus pathophysiology [52,53]. Furthermore, the integration of auditory and limbic information occurs within the MGB [54]. Connected limbic structures, such as the amygdala and nucleus accumbens, are related to emotional and attentional symptoms of tinnitus [55]. Hence, the MGB acts as a central hub in networks involved in tinnitus, which makes it a promising structure for neuromodulatory approaches. 

Currently, MGB DBS has not been applied in humans. The majority of patients with tinnitus can be treated with non-invasive methods, and only a small number of patients can be considered a candidate for DBS. Based on our pre-clinical findings in rat studies, we developed this protocol for a human pilot study.

The primary objective of the proposed study is to assess the safety and feasibility (acceptability) of bilateral MGB DBS in severe tinnitus. Patients with severe tinnitus who are refractory to the standard treatment program will be included. Secondary outcomes will provide data on the potential efficacy of MGB DBS on tinnitus severity (Tinnitus Functional Index (TFI)), tinnitus loudness, and distress (Visual Analogue Scales (VAS)). Additionally, hearing (audiometry), cognition (neuropsychological test battery), quality of life, and psychological functioning (questionnaires) will be assessed. Furthermore, electrophysiological data will assess fundamental aspects of auditory function and tinnitus pathophysiology. After a successful evaluation of the primary and secondary outcomes in this pilot study, MGB DBS could potentially be further developed as a novel treatment option in severe, refractory tinnitus.

## 2. Methods 

### 2.1. Study Design

**Figure 1:**
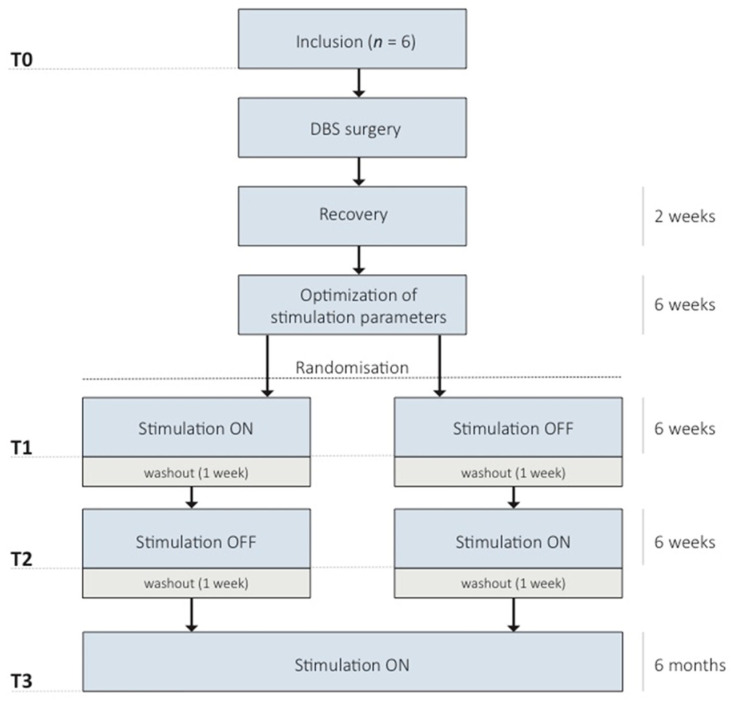
Overview of study design.

This pilot study uses a double-blind 2 × 2 crossover design in which MGB DBS will be compared to no stimulation (Figure 1).

### 2.2. Setting

This study will be carried out at Maastricht University Medical Center (MUMC+) in Maastricht, the Netherlands. MUMC+ is an expertise center for tinnitus, providing integrated multidisciplinary diagnostics and rehabilitation for a wide range of tinnitus patients. The Ear, Nose, and Throat department has long-standing clinical expertise and experience with developing neuromodulative therapies for tinnitus such as intracochlear devices [56]. The neurosurgery department has substantial clinical and preclinical expertise in DBS. In addition to a preclinical DBS research line [57], it is well equipped to conduct clinical trials for new indications of DBS therapy such as Gilles de la Tourette syndrome [58]. Acquisition of neurophysiological data, both intra- and postoperatively is standard practice and is used to unravel neural mechanisms [59]. 

The study will be conducted according to the principles of the Declaration of Helsinki (Version 10, 2013) and in accordance with the Medical Research Involving Human Subjects Act (in Dutch: ‘Wet medisch-wetenschappelijk onderzoek met mensen’ (WMO)). This study complied with the CONsolidated Standards Of Reporting Trials (CONSORT) extension statement. Ethics approval was obtained by the institutional review board. Results will be published in an international peer-reviewed scientific journal and presented at scientific meetings.

### 2.3. Recruitment and Consent

Patients are eligible to enroll if they meet the inclusion and none of the exclusion criteria as outlined in Table 1. All patients will be evaluated and selected by a multidisciplinary team of specialists (otolaryngologists, audiologists, neurosurgeons, neurologists, psychiatrists and psychologists). Diagnostics and treatment are in accordance with national tinnitus guidelines [60]. Based on the Dutch tinnitus guidelines, the tinnitus questionnaire (TQ) is used to determine tinnitus severity [61]. Patients suffer severely (TQ score ≥ 47), and are refractory to available treatments including cognitive behavioral therapy and hearing aids in case of hearing loss.

Patients will be recruited at the outpatient clinic of the Ear Nose Throat department. If patients give permission, they receive an information brochure. Two weeks after, researchers will contact the patients to plan a face-to-face meeting. During this meeting, a full understanding of the study protocol is ensured and additional questions are answered. When a patient needs more time to decide, the investigator plans a follow-up appointment after a few weeks. If a patient agrees to participate in the study, informed consent will be signed by the patient and investigator. If the patient meets the criteria, a second outpatient visit will be planned. During this visit, an intake interview will be conducted by one of the researchers, followed by a consultation with both a psychiatrist and a neurosurgeon. Then, the multidisciplinary team will form a collective decision on inclusion or exclusion. Following a positive decision of the multidisciplinary team, a standard clinical workup for DBS surgery will follow. This includes conducting a brain scan and general blood examination. If inclusion criteria are still met, final inclusion will follow. Patients can leave the study at any time for any reason without any consequences. The investigators can decide to withdraw a subject from the study for urgent medical reasons. 

### 2.4. Outcomes

The time frame and methods of data acquisition are displayed in Figure 1 and Table 2, respectively.

#### 2.4.1. Primary Outcomes

The primary focus of this pilot study is to assess safety and feasibility. Safety will be assessed by reporting the rate and grade of all surgical and stimulation induced adverse events in the study sample during the study period. Feasibility will be assessed in terms of the acceptability of the intervention, by taking qualitative interviews at all major time points (T0, T1, T2, T3; see Table 3), and by comparing satisfaction during sham stimulation and DBS. 

#### 2.4.2. Secondary Outcomes

This pilot study will be used to robustly examine the suitability and appropriateness of the secondary outcome measures (e.g., tinnitus severity, hearing function, depression, anxiety, cognitive function, and quality of life) and make necessary changes in preparation for a full trial. Descriptive statistics and confidence intervals will be reported. The small sample size may hinder a statistically significant outcome. Furthermore, changes in neuronal activity will be assessed by comparing electrophysiological measurements during sham stimulation and DBS.

1. Tinnitus severity will be assessed with the Tinnitus Functional Index (TFI) [62]. The TFI is a validated self-report questionnaire that measures the overall severity of tinnitus and provides coverage of multiple tinnitus severity domains. This questionnaire is the most appropriate responsive measure of treatment-related change. The TFI is translated and validated for Dutch native speakers [63].
2. Tinnitus loudness and burden will be measured by VAS. This will be performed three times daily within a week, which is repeated four times during the study. Furthermore, these VAS scores will be used to assess the effect of stimulation on tinnitus during surgery. VAS ratings for tinnitus loudness and burden are often used in both clinical practice and experimental and descriptive research as a measure of subjective symptoms [64]. Both scales have been shown to correlate with the scores on Tinnitus Questionnaires [65].
3. The hearing function will be assessed with pure tone and speech audiometry. These are clinical standard audiometric tests. Furthermore, subjective hearing will be evaluated using patient feedback.
4. Cognitive functioning will be measured using a validated test battery for standard DBS care. These include the Boston Naming Test, Verbal Fluency, Letter Fluency, 15 Words Test, Trail Making Test part A and B, and the Stroop Color-Word Test.
5. Quality of life and psychological functioning will be assessed by the following psychological questionnaires: 36-Item Short Form Health Survey (SF-36), Beck Depression Inventory II (BDI-II), Beck Anxiety Inventory (BAI), and Hospital Anxiety and Depression Scale (HADS).
6. Neurophysiological measurements: electrophysiological data and auditory brainstem responses will be recorded before and after surgery (T0 and recovery) and at the end of treatment periods I and II (T1 and T2). Furthermore, local field potentials (LFP) will be recorded during surgery and before the implantation of the pulse generator. Details are described under ‘Neurophysiological assessments’.

Dependent on the results of the primary and secondary outcomes, future implementation procedures might be adapted or in case of adverse events, terminated. These adaptations depend not only on the feedback of the individual participants but also on expert judgment. For example, in case the patients describe the questionnaire procedures to be too burdensome, it could be considered to shorten these. Another example, based on expert judgment, could be rethinking the two times six weeks crossover design in case no short-term effects are reported. 

## 3. Intervention

### 3.1. Implantation of DBS Electrodes and Internal Pulse Generator

A two-staged surgery will be performed. First, bilateral DBS electrodes will be implanted. Tinnitus is a complex disorder for which to date the underlying mechanisms are not entirely clear. The auditory system is organized bilaterally, with a large number of interconnections and information crossing partially (80%) after the cochlear nucleus. To investigate the safety and feasibility bilateral implantation was opted for. Following a standard stereotactic surgical procedure, DBS electrodes will be inserted in the MGB of the thalamus and monitored by radiological and electrophysiological measures. The placement will be conducted under local anesthesia. A CT cerebrum will be carried out and fused with a pre-operative MRI in order to plan the exact trajectory. First, a micro-electrode (InoMed, Emmendingen, Germany) will be inserted. Neurophysiological recordings will be performed in 0.5 to 1 mm steps from 10 mm above and maximally 5 mm below the target with a multi-channel system (InoMed, Emmendingen, Germany) [66]. Simultaneously, at each step, a sequence of auditory stimuli will be presented, which is designed based on known signal processing characteristics of the thalamus [67] and to maximize the likelihood of evoking a reliable response. The amplitude of these responses relative to the spontaneous activity will be used to confirm electrode placement. After the identification of the ventral and dorsal border of the MGB, test stimulation will be applied using 130 Hz, a pulse width of 120 µs and a voltage starting from 0.5 V up to 5 V, or until undesired side effects occur. Stimulation is monopolar, the deepest contact of the electrode is the anode and the battery is the cathode. Stimulation amplitude will be adjusted stepwise. The patient will be asked repeatedly to rate the loudness and burden of the tinnitus sound using VAS. Furthermore, the neurologist will test for undesired side effects. The stimulation electrode will be placed once an optimal effect and acceptable or absent side effects are reached. After placement and fixation of both stimulation electrodes (Medtronic, model 3389) the stereotactic frame will be removed and electrodes will be externalized to enable the recording of LFPs postoperatively. 

After surgery, the patient will be transferred to a neurosurgical medium care unit. On the second post-operative day a CT cerebrum will be made to confirm the definite electrode positioning. After one or two days the pulse generator (Medtronic, Activa PC model 37601) will be subcutaneously placed under general anesthesia and the electrodes will be connected. One or two days postoperative, the patient can be discharged. After the end of the trial, follow-up as standard DBS care will be provided. 

### 3.2. Stimulation Parameters

Based on the results in rats it is expected that only high-frequency stimulation will be effective for tinnitus reduction [43]. The following stimulation parameters will be used as the minimum and maximum values during the optimization period to determine optimal stimulation parameters: stimulation frequency (2–200 Hz), pulse width (60–450 μs), and voltage (starting from 0 to a maximum of 5 V). Initially, the stimulation will be bilateral with the same parameters on both sides. Voltages will be increased symmetrically depending on the clinical effect and side effects. In case of stimulation induced side effects, bilateral stimulation can be adapted to unilateral stimulation. During treatment, patients will visit the outpatient clinic weekly. During this period, side effects and tolerability of the stimulation in daily life are evaluated; however, no instant change in tinnitus is expected due to the complexity of the disorder and the anticipated burden and duration of tinnitus. Following the treatment episode, there will be a one-week period for washout of a possible residual therapeutic effect. 

### 3.3. Neurophysiological Assessments

EEG will be recorded with surface electrodes applied to the scalp in accordance with the 10–20 international system standard. Just before the implantation of the pulse generator, cables are externalized, which enables simultaneous recordings of LFPs from the depth electrodes in the MGB. 

For each session, several recordings with and without auditory stimulation will be obtained. In the first phase, resting-state activity will be recorded with eyes open and eyes closed. These initial recordings serve to establish baselines for task-independent neural oscillatory activity, network activity, and coherence (assessed on the basis of dominant spontaneous low-frequency oscillations and their variability). In the second phase, auditory brainstem responses will be recorded using a standard protocol to probe auditory brainstem function before and after surgery. In the third phase, activity will be recorded during experimental auditory stimulation. The respective measures will allow assessing of basic characteristics of auditory function in general, and sensory gating in particular (i.e., adaptive filtering based on predictable feature-based and temporal information) in accordance with the adopted model. These measures have been previously obtained via surface EEG recordings in humans [68,69] and preliminary data show comparable responses recorded from the thalamus in the animal model.

Taken together, these measures allow for assessing fundamental signal coding (linear vs. event-related), time-course (temporal relation of thalamic and surface responses), and functional (deviance processing, regularity, predictability, gating) aspects of auditory function. Dysfunctional processing would correspondingly be indexed by desynchronization, lack of suppression effects, temporal decoupling (reduced correlation between thalamic and surface responses), and overall high variability. 

### 3.4. Randomization and Blinding

Randomization will be performed directly after the period of stimulation optimization by an independent institution, the Clinical Trial Center Maastricht (CTCM). Patients will be randomly assigned to one of the two study groups. The investigator who adjusts DBS parameters cannot be blinded. All other investigators and the patient are blinded to the study group. In case the patients are considered unblinded due to the nature of the stimulation the protocol will be carried out as planned. Only in the case of medical concerns, the patient and investigators will be unblinded in order to provide the care needed.

### 3.5. Data Collection and Management

Data handling will be organized according to the “FAIR guiding principles for scientific data management and stewardship” [70]. This will be carried out in cooperation with the CTCM, which is a local center that facilitates human research. Data will be collected by data entry in Castor electronic data capture (EDC); a cloud-based, password-protected data management system providing a backup on a secured server. Audit trailing provides basic data to backtrack a file to its origin. Paper versions of questionnaires will be kept in a locked closet in the research office. All data will be anonymously coded. The key will be available to the project leader only. Data collection is monitored by the CTCM; a specific monitor is appointed to the study who will follow up on the progress and adherence to the protocol. The monitor will perform periodic checks. A data safety monitoring board, comprising a statistician and two neurologists, is instituted which periodically reviews and evaluates the accumulated study data concerning participant safety, study conduct, progress, and efficacy. The data safety monitoring board receives and reviews the progress and acquired data of this trial and provides the research team with advice on the conduct of the trial.

### 3.6. Statistical Analyses

The safety and feasibility outcomes will be reported descriptively. Descriptive statistics with 95% confidence intervals will be used to present preliminary data of secondary outcomes such as tinnitus (severity, loudness and distress), anxiety, depression, hearing function, quality of life, and cognitive functioning. These data will provide some insight into population characteristics and might indicate potential changes in mean scores between the intervention and sham stimulation.

### 3.7. Sample Size

As this is a pilot study, no formal sample size calculation was performed. Only a small sample size will be used because a large burden is being placed on the participants. Additionally, there is uncertainty about potential benefits and the fact that the effects of surgery are still unknown, even though it is expected to be safe. Based on previous first-in-human DBS studies [24,58,71], we expect to adequately address the safety and the proof of concept purpose of the study by including six patients. In these invasive first-in-human trial small sample sizes are not uncommon. Furthermore, this number of patients will enable the collection of preliminary results that will provide meaningful information about the differences between the intervention and sham stimulation. In case of the withdrawal of a patient or in case of incomplete data there will be no replacement of individual participants.

### 3.8. Patient and Public Involvement

Patients from the Dutch tinnitus support group were involved in the development of this protocol. During information meetings, aspects of the study were discussed such as feasibility and eligibility criteria. Furthermore, patients were involved in the development of patient information. 

## 4. Discussion

One of the main ethical considerations in this study is balancing risks and benefits. The potential benefit of the intervention is a reduction of tinnitus loudness and tinnitus burden. Risks of this minimally invasive and reversible form of functional neurosurgery are surgery-related complications (e.g., cerebral hemorrhage or infarction, CSF leak, seizures, meningitis or encephalitis), hardware failure (e.g., lead rupture, extension fibrosis, device migration) and stimulation related side effects. These latter effects are unknown as clinical MGB DBS has not been performed before. The function of the MGB is primarily hearing thus other side effects than hearing-related effects are less likely. Animal studies did not show hearing loss, anxiety or locomotion-related side effects in DBS in subcortical auditory structures [43,47,48]. We also reviewed possible side effects based on the brain structures surrounding the MGB. The MGB is located posterior to the subthalamic nucleus. The MGB is 8mm wide, 6mm long and 6.5mm high with its largest volume at -3.5mm from the AC-PC (anterior commissure-posterior commissure) plane. Considering the relatively large size of the MGB, current spread outside the MGB is unlikely if the DBS electrode is positioned in the center. Neighboring structures of the MGB are structures similar to other commonly used stimulation targets. The current spread to the anterior side of the MGB could result in internal capsule effects. These side effects are known from subthalamic nucleus (STN) DBS treatment for dystonia and Parkinson’s disease. The possible side effects of internal capsule stimulation are dysarthria, muscle contractions, and gaze paresis. Posterior to the MGB the ventricle can be found. Possible side effects are known from anterior nucleus of the thalamus (ANT) DBS which has also a border at the ventricle. Antero-medially the sensory thalamus is located which is targeted when performing DBS to treat pain. More medially the fields of Forel are located. Possible side effects are known from STN DBS in which current spread also may occur to the fields of Forel which can result in disturbances of speech, postural stability and gait. Laterally to the MGB the optic tract is located. We know from globus pallidus internus (GPi) DBS that stimulation of the optic tract elicits phosphenes. All these side effects from current spread to surrounding regions are elicited by stimulation and thus reversible. Taking this together, MGB DBS could induce changes in auditory sensation, and there is a slight risk for side effects due to current spread. In case of undesired side effects, stimulation parameters can be adapted or stimulation can be turned off. The principal investigator will immediately inform the data safety monitoring board and the medical ethical committee in case of serious side effect. 

In relation to correctly and carefully evaluating risks and benefits, patient recruitment and extensive informed consent are crucial steps. The primary inclusion criterion is that patients need to be refractory to current treatment (e.g., cognitive behavioral therapy); second, sufficient hearing is required and patients cannot be candidates for cochlear implants. In a refractory patient population, patient selection is an important factor. Patients should have gone through all available proven treatments without significant success. This treatment could be the last resort for these patients when all else has failed. However, this could also affect their decision-making, making them eager to participate in the hopes of a cure. Therefore, patients should be informed about the implications and the uncertainties concerning the outcome extensively during the informed consent procedure. Thus, creating realistic outcome expectations and emphasizing the potential effects or the potential absence thereof. To evaluate whether a patient will be able to cope with a potential negative outcome (no result or side effects) a multidisciplinary team is involved in this study. Additionally, both the potential health benefits as well as the scientific gain need to outweigh the possible risks. In order to maximize the scientific benefit one should try to gather as much information as possible to expand the fundamental knowledge. The potential health benefit should be aimed at increasing the participant’s quality of life. For this reason, a balance needs to be struck to obtain the most beneficial situation for both science and the participant [72]. 

A questionnaire study showed that about 20% of general tinnitus patients are willing to undergo DBS surgery in case of a 50% chance of successful treatment. The willingness increased with the number of therapies already tried [73]. Further, patients would be willing to pay 20 times their monthly income to be treated. Most patients would accept a risk of mild side effects, and almost half of the patients would accept a risk of severe side effects [73]. A caveat in patient selection is that desperate patients might see this experimental treatment as a last resort and rush through the informed consent [74]. These circumstances make this group of patients vulnerable and inclusion should carefully be contemplated when informed consent is discussed. Included patients will receive a heavy burden by undergoing a surgical treatment that could be perceived as a last remedy. This could potentially lead to a bias based on effort justification. We aim to minimize the bias by using a combined crossover and randomized double-blind design. The crossover ensures that every patient receives each treatment in a different order, counterbalancing the treatment phases. The double-blind randomization ensures that neither the participant nor the researcher can influence the outcome as they are unaware of the condition. The rationale of the study is for the majority based on animal studies. The assessment of tinnitus in rats (GPIAS) and the method for DBS is at least not fully translatable to humans. An additional limitation is that generalizability to the target group of severely affected tinnitus patients might be challenging due to the heterogeneous nature of tinnitus and the small sample size of this pilot feasibility trial. Still, by carefully evaluating safety and feasibility in this pilot study, we will be able to determine how MGB DBS is received by participants, and optimize a follow-up study in terms of patient support, patient inclusion, surgical procedure and choice of stimulation parameters.

## 5. Trial Status

Recruitment starts in 2020 and last follow-up is estimated to be completed in 2023.

## Figures and Tables

**Table 1:** Overview of preclinical and clinical DBS studies that applied DBS primarily for tinnitus. We only listed studies that primarily treated tinnitus. GPIAS = gap-prepulse inhibition of the acoustic startle reflex; TFI = Tinnitus Functional Index (clinically significant change, i.e., responder = ≥ 13 point decrease); THI = Tinnitus Handicap Inventory (clinically significant change, i.e., responder = ≥ 20 point decrease).

Animal (Rodent) Studies						
Reference	Design	Target	N	Uni/Bilateral	Stimulation	Outcome
Van Zwieten et al., 2019 [43]	Noise induced tinnitus, within-subject controlled	Dorsal Cochlear Nucleus	10	Bilateral	Continuous stimulation during test	GPIAS, tinnitus behavior was suppressed
Van Zwieten et al., 2018 [47]	Noise induced tinnitus, within-subject controlled	Medial Geniculate Body	11	Bilateral	Continuous stimulation during test	GPIAS, tinnitus behavior was suppressed
Ahsan et al., 2018 [45]	Noise induced tinnitus, within-subject controlled	Dorsal Cochlear Nucleus	9	Bilateral	Continuous stimulation during test	GPIAS, tinnitus behavior was suppressed
Smit et al., 2016 [46]	Noise induced tinnitus, within-subject controlled	Inferior Colliculus	9	Bilateral	Continuous stimulation during test	GPIAS, tinnitus behavior was suppressed
Luo et al., 2012 [44]	Noise induced tinnitus, within-subject controlled	Dorsal Cochlear Nucleus	6	Unilateral	Continuous stimulation during test	GPIAS, tinnitus behavior was suppressed
**Human Studies**						
**Reference**	**Design**	**Target**	**N**	**Uni/bilateral**	**Stimulation**	**Outcome**
Cheung et al., 2019 [24]	Open-label, nonrandomized trial in refractory tinnitus patients	Caudate Nucleus	6	Bilateral	24 weeks open label	TFI (3 responders), THI (4 responders)
Dijkstra et al., 2018 [25]	Case report in refractory tinnitus patients	Ventral anterior limb of the internal capsule & Nucleus Accumbens	1	Bilateral	1 year	TFI (pre = 74, post = 46), THI (pre = 76, post = 32)

**Table 2:** Eligibility criteria. TQ, Tinnitus Questionnaire; DBS, Deep Brain Stimulation; CI, Cochlear Implant; ABI, Auditory Brainstem Implant.

Inclusion Criteria	Exclusion Criteria
Medically refractory tinnitus * Age 18–69 years Experiencing tinnitus that is non-pulsatile and uni- or bilateral Severe tinnitus (TQ score ≥ 47) Tinnitus, which is chronic (present ≥ two years) and stable (not intermittent ≥ one year) Average pure tone thresholds for 1, 2 and 4 kHz <60 dB for each ear Willingness to participate in the study	Anatomic cause of tinnitus (e.g., vestibular schwannoma, tumor, middle-ear pathology, temporal mandibular disorder) DSM-V psychiatric disorders, other than depression or anxiety disorder Depression or anxiety disorder, manifestation before tinnitus onset Cognitive impairment or coping problems Active otologic diseases Pregnancy or breast-feeding Active suicidal thoughts or recent attempts Life expectancy lower than two years Implantable electronic devices that potentially interfere with DBS (CI, ABI, cortical implant) General contra-indications for MRI or surgery

* Patient does not respond to available tinnitus treatments (e.g., sound enrichment and cognitive behavioral therapy) and is thoroughly evaluated by the multidisciplinary tinnitus team in MUMC+.

**Table 3:** Schedule of assessments and procedures. T0 is at inclusion. T1 is at the end of the first 6-week treatment period (stimulation ON or OFF). T2 is at the end of the second 6-week treatment period (stimulation OFF or ON). T3 is at the end of the third treatment period of 6 months stimulation ON. MRI, Magnetic Resonance Imaging; CT, Computer Tomography; TFI, Tinnitus Functional Index; VAS, Visual Analogue Scale; ABR, Auditory Brainstem Responses; EEG, Electroencephalography; LFP, Local Field Potentials.

		Inclusion (T0)	Surgery	Recovery	Optimization	Period I (T1)	Washout	Period II (T2)	Washout	Period III (T3)
**Visits and Procedures**										
Number of Outpatient visits		2			6	1		1		1
Anesthesiology screening		•								
MRI		•								
CT			•	•						
**Outcome Measures**										
Tinnitus severity	TFI	•			•	•		•		•
Tinnitus loudness and burden:	VAS *	•	•		•	•		•		•
Hearing function:	Audiometry ABR	•		•		•		•		•
		•		•		•		•		•
Cognitive functioning **		•				•		•		•
Psychological functioning ***		•				•		•		•
Neurophysiology:	EEG LFP	•		•		•		•		
			•	•						

* Last week of the period, 3 random times per day. ** Cognitive functioning tests include Boston Naming Test, Verbal and letter fluency, 15 Word Test, Trail Making Test and Stroop Color-Word Test. *** Psychological functioning is assessed using 36-Item Short Form Health Survey (SF-36), Beck Depression Inventory II (BDI-II), Beck Anxiety Inventory (BAI), and Hospital Anxiety and Depression Scale (HADS).

## Data Availability

Data can only be viewed by the investigators, IGJ and monitors.

## List of Abbreviations

DBS	Deep brain stimulation
MGB	Medial geniculate body
TFI	Tinnitus Functional Index
VAS	Visual Analogue Scales
MUMC+	Maastricht University Medical Center
TQ	Tinnitus questionnaire
SF-36	36-Item Short Form Health Survey
BDI-II	Beck Depression Inventory II
BAI	Beck Anxiety Inventory
HADS	Hospital Anxiety and Depression Scale
LFP	Local field potentials
CTCM	Clinical Trial Center Maastricht
EDC	Electronic Data Capture
STN	subthalamic nucleus
AC-PC	Anterior Commissure-Posterior Commissure
ANT	Anterior Nucleus of the Thalamus
GPi	Globus Pallidus Internus
